# Supplementary material for: Reliability of Different Anterior Cranial Base Reference Areas for Voxel-Based Superimposition
Source: J Clin Med. 2021 Nov 20;10(22):5429. doi: 10.3390/jcm10225429 (PMC8622398; doi:10.3390/jcm10225429)
Supplement: Supplementary file 1 [file jcm-10-05429-s001.zip › jcm-1448414-supplementary.pdf]

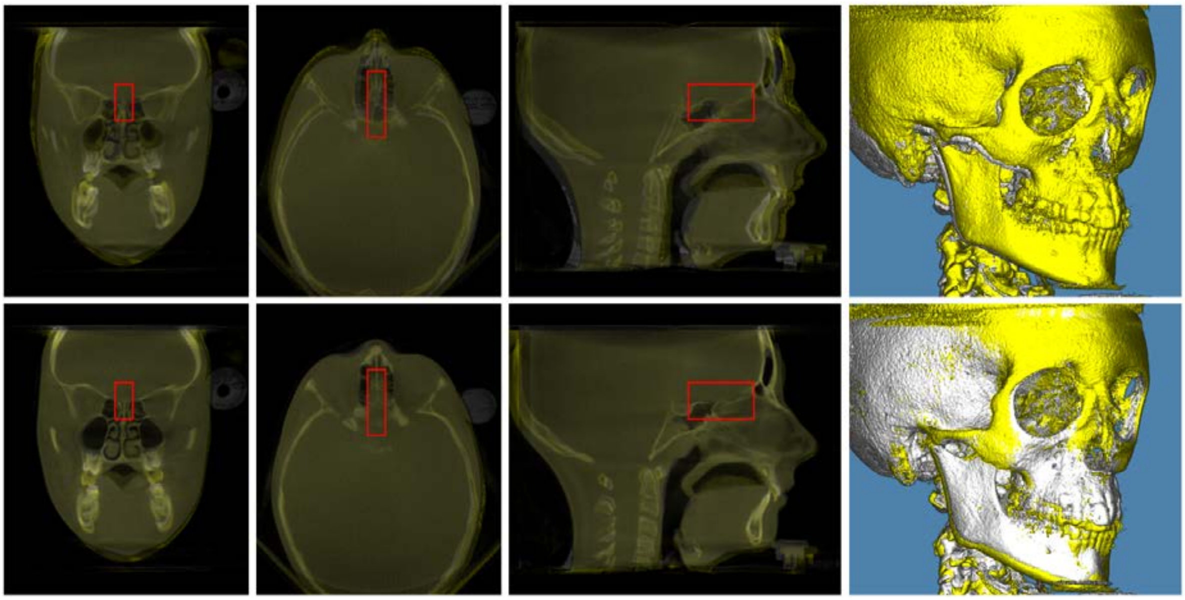

Figure S1. Voxel-based superimposition on the middle anterior cranial base (MACB) in Dolphin 3D Software. The red frame in the cranial, axial, and sagittal view represents the selected superimposition reference area. The MACB selection frame included the posterior wall of the sinus frontalis (anteriorly) and the middle of the sella turcica (posteriorly) and was set inferiorly 2–4 mm below the lowest point of sella turcica. The superior limit was located 1 cm above the anterior clinoid process. Additionally, the lateral extension of the frame was delimited by the width of the anterior wall of sella turcica. Pre (**above**) and post (**below**) images depict the repositioning of the CBCT T1 image according to the position of CBCT T0.

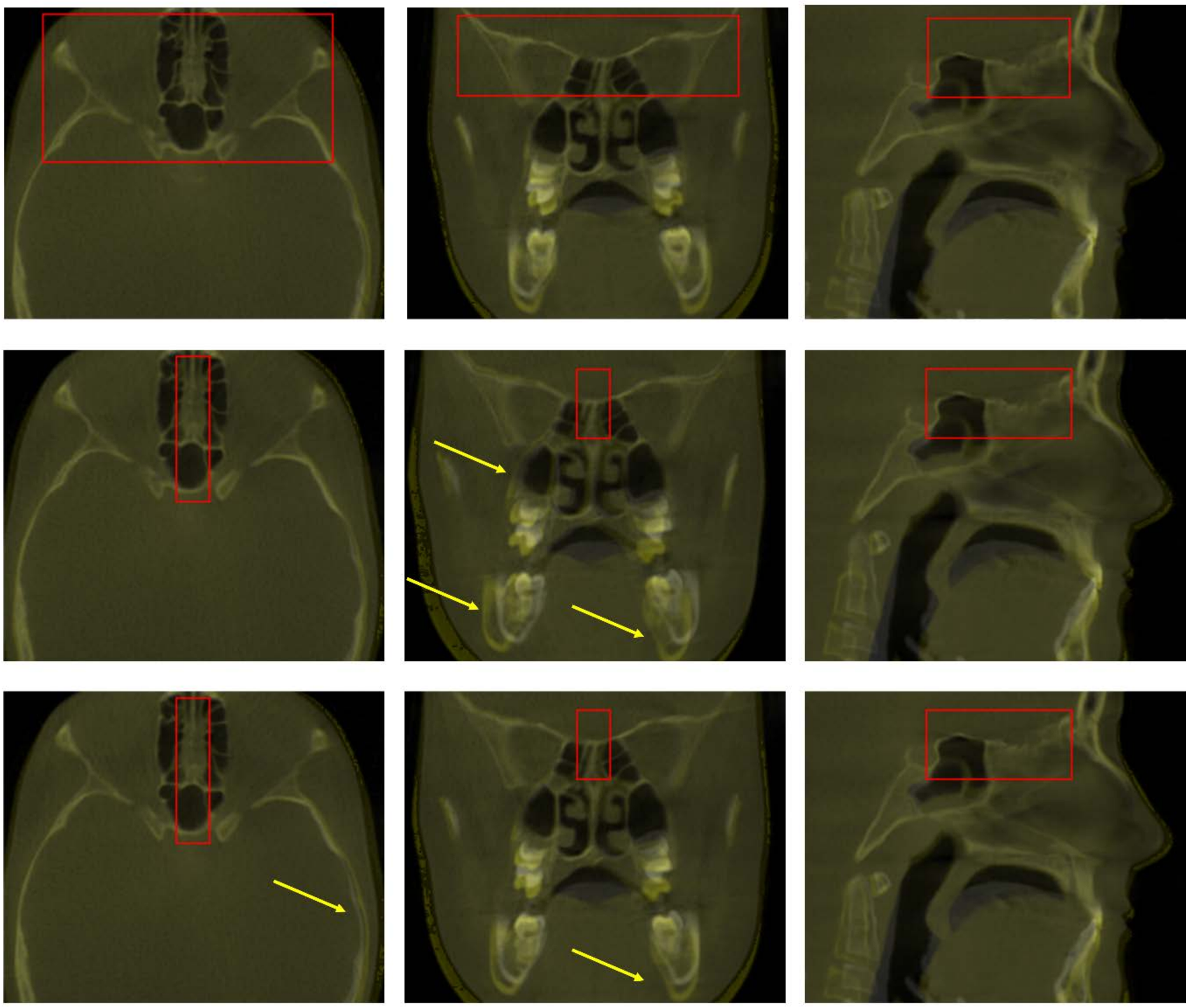

Figure S2. Three repeated superimpositions of the same case using the total anterior cranial base (TACB) reference area (top row) and the middle anterior cranial base (MACB) (middle and bottom rows) reference area. Adequate superimposition is shown in the sagittal dimension in all cases; however, the axial and coronal dimensions show differences in overlap (yellow arrows). Note the large discrepancy between the repeated superimpositions on the MACB reference area.
